# Self Assessment Memory Scale (SAMS), a new simple method for evaluating memory function

**DOI:** 10.3389/fnagi.2022.1024497

**Published:** 2022-11-22

**Authors:** Hisatomo Kowa, Maki Uchimura, Asuka Ohashi, Mamoru Hiroe, Rei Ono

**Affiliations:** ^1^Graduate School of Health Sciences, Kobe University, Kobe, Japan; ^2^Graduate School of Medicine, Kobe University, Kobe, Japan; ^3^Department of Physical Activity Research, National Institutes of Biomedical Innovation, Health and Nutrition, Tokyo, Japan

**Keywords:** Wechsler Memory Scale (WMS), MCI, Alzheimer's disease, delay recalling, self assessment

## Abstract

We have developed a new method for easy self-assessment of changes in memory recall impairment, which can be used during the very early stages of dementia. An 8-picture recall and a 16-word regression were assessed, respectively, and the index was calculated by adding up the ratio of correct responses to both tests. A total of 85 subjects including 12 MCI, 8 AD, and 65 older persons with normal cognitive function were evaluated, and the correlation with the WMS-R Logical Memory II score was examined. The results showed that there was a statistically significant correlation between the 8-picture recall (*R* = 0.872, *p* < 0.0001) and the index (*R* = 0.857, *p* < 0.0001), respectively, with the Logical Memory score. We have named this index as Self Assessment Memory Scale (SAMS), and are now developing a digital tool to enable easy and self-administered evaluation of recall.

## 1. Introduction

Alzheimer's disease (AD) affects around 47 million people worldwide and is predicted to affect 135 million people by 2050 (Ricci, 2019). Developed countries such as Japan, Italy, Germany, Portugal, Greece, France, and Spain, which are all super-aging societies, have the highest rates of dementia disease per 1,000 people, and more than one in 25 people will be living with dementia in four of these countries (Japan, Italy, Portugal and Spain) (OECD, 2019). Pharmaceutical companies throughout the world are working to develop a treatment, and in 2021, aducanumab became the first Alzheimer's disease modifying agent to be approved by the FDA (FDA, 2021). Aducanumab showed a 22% reduction in the decline of the Clinical Dementia Rating Sum of Boxes (CDR-sb) (Cummings et al., 2021), indicating relatively limited effectiveness and no regain of memory function following the onset of the decline. For this reason, the target period for disease-modifying treatments has shifted to the preclinical stage, when brain pathology has begun to change but clinical symptoms have not yet clearly emerged. In order to enable early intervention for patients during the preclinical stage, there is a critical need for a system or tool for the early detection of slight changes in cognitive functions that can be performed in a short time and outside hospital settings. The earliest cognitive change in Alzheimer's disease is impaired memory function, and the logical memory subtest of the Wechsler Memory Scale revised version (WMS-R) has been used in ADNI and other studies for this purpose. In particular, the delayed recall score of a narrative sentence after 30 min (called logical memory II, LM II) is applied for detecting amnesic mild cognitive impairment (Iwatsubo et al., 2018). The challenges associated with this test are that it requires more than 30 min to perform and the scoring step is complicated. Other tests for memory include word recall and recognition, which are used in conjunction with the Alzheimer's Disease Assessment Scale-Cognitive Subscale (ADAS-Cog) (Rosen et al., 1984), the Free and Cued Selective Reminding Test (FCSRT) (Grober et al., 2000; Lemos et al., 2015), the Montreal Cognitive Assessment (MoCA) (Nasreddine et al., 2005), and other scales. In addition to the conventional test methods described above, which are conducted by a licensed clinical psychologist, new digital cognitive tests, such as Cogstate (Maruff et al., 2013), Mild Cognitive Impairment Screen (MCIS) (Shankle et al., 2005), and CogEvo (Ichii et al., 2020) have become commercially available; however, they are not suitable for early detection of cognitive decline because these digital tools do not sufficiently weight assessment of memory functions. In light of this situation, we have conducted a study to establish a new method for memory evaluation that shows a good correlation with the LM II subtest score of the WMS-R and can also be conducted easily in a short time.

## 2. Materials and methods

### 2.1. Participants

The subjects of this study were divided into three groups: Alzheimer's disease (AD) and patients with mild cognitive impairment (MCI) diagnosed clinically based on the NIA/AA Guidelines for Diagnosis of AD (McKhann et al., 2011) and MCI (Albert et al., 2011) at the Department of Neurology, Kobe University Hospital, and healthy volunteers living in senior housing in Kobe City or recruited through websites. The sample sizes required to calculate each correlation coefficient when α = 0.05 for statistical purposes and 0.9 for power are 38 cases for *R* = 0.5, 25 cases for *R* = 0.6, and 17 cases for *R* = 0.7. Overall, we analyzed data from 8 patients with AD (Male: 83.1 ± 0.0, Female: 79.9 ± 4.6 years), 12 patients with MCI (Male: 75 ± 4.0 years, Female: 77.5 ± 5.5 years), and 65 controls (Male: 72.6 ± 8.8 years, Female: 71.9 ± 8.7 years). Written informed consent was obtained from all subjects, and in the case of the AD/MCI group, from their guardians. Table 1 shows the subject demographics.

**Table 1:** Subject demographics.

	**Control**		**Mild cognitive** **Impairment (MCI)**		**Alzheimer's disease (AD)**	
Number	65		12		8	
Sex	M:33	F:32	M:6	F:6	M:1	F:7
Age(SD)	72.6 ± 8.8	71.0 ± 8.7	75.0 ± 4.0	77.5 ± 5.5	83.0 ± 0.0	79.9 ± 4.6
Education (less 9 years)	0	0	1	2	0	1
(less 12 years)	12	16	3	4	1	4
(less 16 years)	18	15	1	1	0	2
(more 16 years)	3	1	0	0	0	0

### 2.2. Method

#### 2.2.1. Preliminary evaluation tasks

Currently, there are numerous well-validated measures available to aid clinicians in the detection of dementia (Ismail et al., 2010). We designed a new method that correlates with the LM II subtest score of WMS-R, because the LM II score is a gold standard method for memory assessment, and is widely used for the diagnosis of MCI. The free verbal recall is generally higher for items presented as pictures than for items presented as words (Paivio and Csapo, 1973), and we applied not a word-presenting but a picture-presenting method. In the primary task, we conducted the LM II task of WMS-R in 30 healthy volunteers first and then conducted word recall (5 words), word recognition (12, 16 words), and then picture recall (8 words) on weekly basis for a total of four sessions. In order to control for learning effects, the words and their order were randomly shuffled for each session. The specific contents of each task are as follows.

##### 2.2.1.1. Word recall task

The words are shown on flashcards sequentially every 3 s and the subjects are asked to read the words out loud and memorize them. The words are all from different categories (vehicles, animals, flowers, parts of the body, household appliances, etc.). After displaying five flashcards, an interrupt task is conducted (ask the subject what she/he had for lunch, interrupt duration: 75 s). Then, the subject is asked to say the words he/she could remember. We adopted this method because the memory task in the MoCA memory test has five words and is considered more difficult than the MMSE memory test that has three words (Fukuda, 2020).

##### 2.2.1.2. Word recognition task

The words are shown on flashcards sequentially every 3 s and the subjects are asked to read the words aloud and memorize them. The words are all from different categories (vehicles, animals, flowers, parts of the body, household appliances, etc.). After displaying a specific number of flashcards (6, 8, or 12), an interrupt task was conducted (ask the subject where the school she/he went to is located, interrupt duration: 75 s). Afterward, several of the previously shown flashcards are presented as well as some dummy words sequentially. Subjects were asked to answer “yes” if the word was shown previously, or “no” if not shown. The rationale for a specific number of cards was based on the memory test of the ADAS-J (10 words). The utility of the ADAS-cog and its subtests are optimal in the moderate range of cognitive dysfunction, but raw score differences in that region correspond to relatively small differences in cognitive dysfunction (Benge et al., 2009). Thus, our study focuses on 12 and 16-word numbers.

##### 2.2.1.3. Picture recall task

A picture is shown on a flashcard every 3 s sequentially and the subject is asked to name the picture out loud and memorize it. The pictures are all from different categories (vehicles, animals, flowers, parts of the body, household appliances, etc.). After an interrupt task (ask the subject what she/he had for lunch, interrupt duration: 75 s), the subject is asked to name the pictures previously shown.

#### 2.2.2. Main evaluation tasks

In the main task, all 85 subjects underwent the LM I and II test of WMS-R, an 8-picture recall test, and a 16-word recognition task. All the tasks were conducted on the same day in order to ensure that the correlation between the LM II score and new task results was clear. The correlation between the score on the LM II and that of the 8-picture recall test and 16-word recognition tasks was examined, respectively.

##### 2.2.2.1. 8-picture recall task

**Figure 1:**
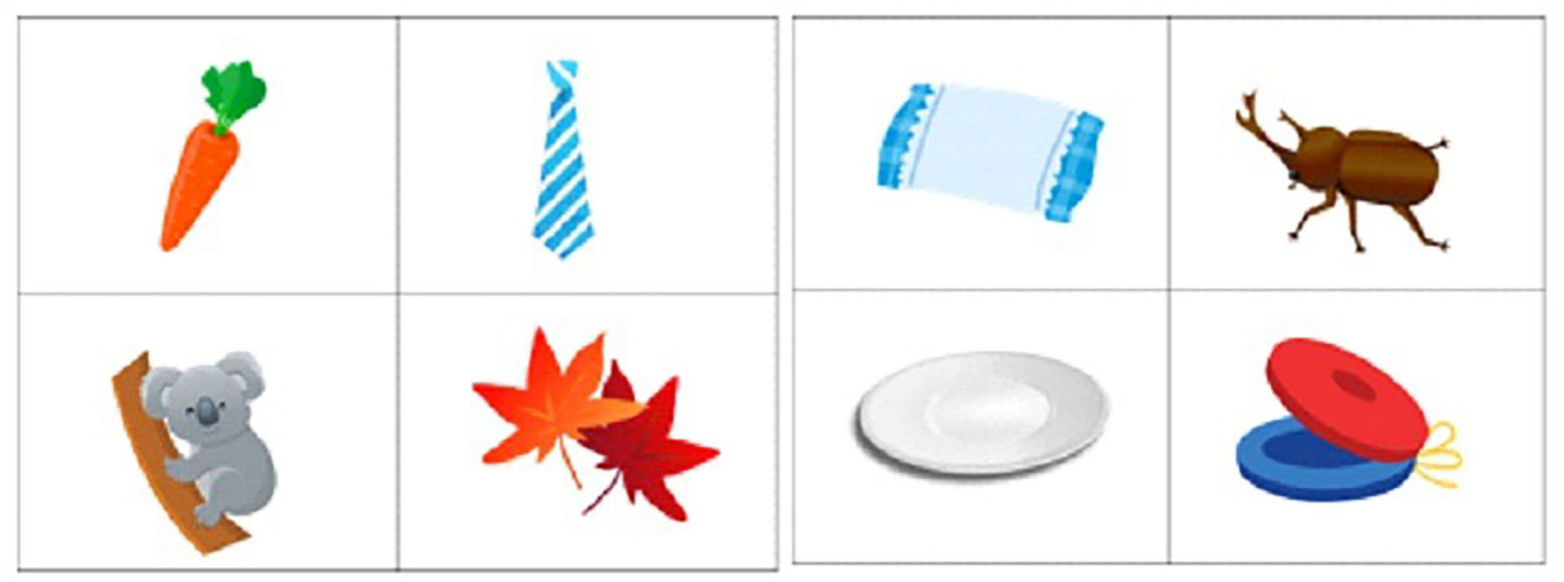
The illustrations on 8 flashcards.

Eight-picture flashcards are shown sequentially every 3 s and then the subjects are asked to name the picture out load and memorize it. Possible interpretations of this recall task includes the superiority of nonverbal imagery as a memory code, and dual encoding favoring pictures (Paivio and Csapo, 1973). All the pictures are from different categories (vehicle/animal/flower/part of the body/appliances, etc.). After 75-s interruption, the subject is asked to name the pictures she/he remembers without any hints Figure 1.

##### 2.2.2.2. 16-word recognition

**Figure 2:**
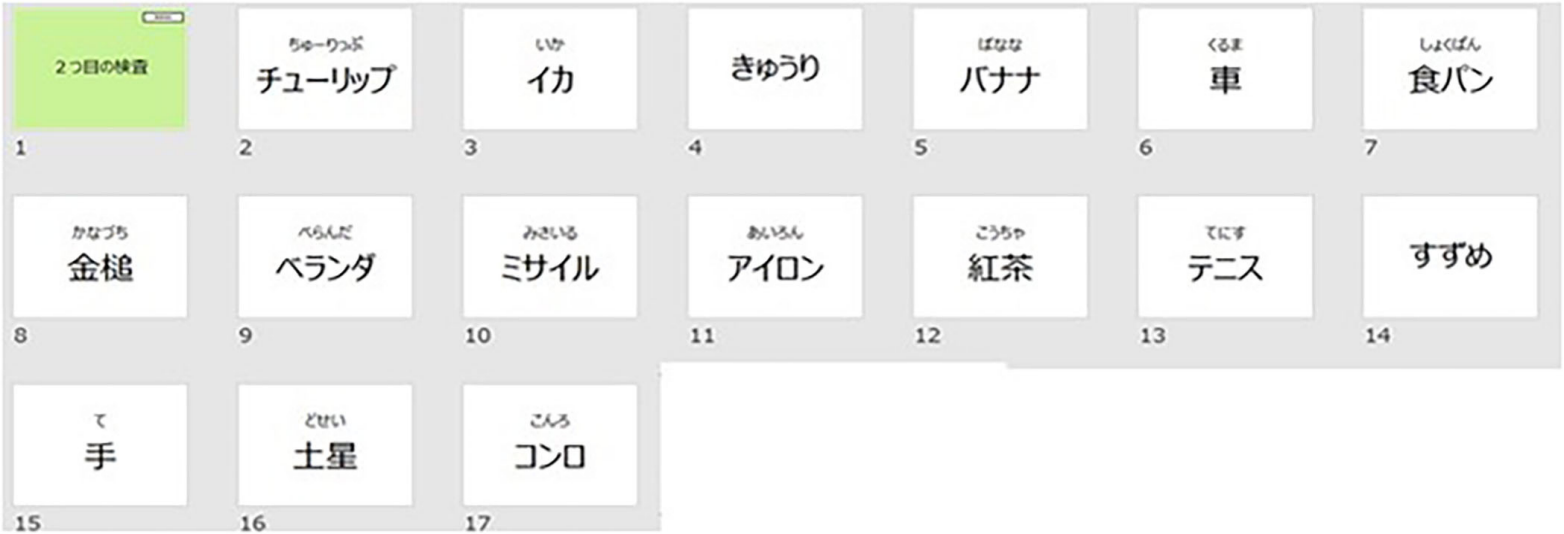
The words shown here in Japanese are as follows, starting from the top left; tulip, squid, cucumber, banana, car, bread, hammer, balcony, missile, iron, autumn leaves, tennis, sparrow, hand, Saturn, and stove.

Sixteen flashcards are shown sequentially every 3 s and the subjects are asked to read the words aloud and memorize them. The words are all from different categories (vehicles/animals/flowers/parts of the body/appliances, etc.). After a total of 16 flashcards are shown, several flashcards are presented sequentially with a hint, including a dummy flashcard word not presented before. The subjects are asked to answer “yes” if the word was previously presented, and “no” if it is not Figure 2.

##### 2.2.2.3. Index of 8-picture recall and 16-word recognition as SAMS (Self Assessment Memory Scale)

In the preliminary task, some results showed a higher correlation with the LM scores of the WMS-R when we integrated two test data instead of a single test. Therefore, we examined the relationship between the LM-II score and an index that added the ratio of correct responses to the 8-picture recall and that of 16-word recognition task, which counts a maximum of 2.0 for the percentage of correct answers.

### 2.3. The statistical analysis

The simple regression analysis was conducted using the following software. SAS JMP Software Version 15.1 for Windows. Python Version 3.8.1.

### 2.4. Ethics review committee

This study was approved by the Ethics Committee of the Graduate School of Health Sciences, Kobe University, and the Ethics Committee of the Graduate School of Medicine, Kobe University. Each subject gave informed consent in accordance with the Helsinki Act.

## 3. Results

### 3.1. Preliminary task results

The results of the primary task studying the relationship between WMS-R LM II score and four recall tasks including word recall (5 words), word recognition (12, 16 words), and picture recall (8 pictures) showed that there was relatively good correlation and reproducibility for the 16-word and 8-pictures tasks Table 2.

**Table 2:** The *R*-value of the correlation results of WMS-R LM II score and four tasks.

	**5-word recall**	**12-word recognition**	**16-word recognition**	**8-picture recall**
1st week	0.07	0.29	0.47	0.44
2nd week	0.08	0.33	0.43	0.27
3rd week	0.16	0.35	0.49	0.31
4th week	0.17	0.27	0.44	0.40
Average	0.12	0.31	0.46	0.36

In addition, when we integrate the two test data, instead of a single test of each, the two integrated result data of 8-picture recall and 16-word recognition showed the highest correlation with the LM II score of WMS-R (*R* = 0.606).

Because of the results from the preliminary task evaluation, the 8-picture recall and 16-word recognition were selected for the main task evaluation with all the healthy volunteers, then MCI and AD subjects. In addition, the index score obtained as the sum of the percentage of correct answers in the two tests (8-picture recall and 16-word recognition) was examined.

### 3.2. Main task results

The results of the main task studying the relationship between WMS-R LM II score and 8-picture recall and 16-word regression are shown in Table 3. For each task, two analyzes were conducted, one with all 85 participants and the other with 65 participants in the control group.

**Table 3:** *R*-value and *p*-value of the correlation between WMS-R LM II score and each new methods of this study.

		**8-picture recall**	**1 6-word recognition**	**SAMS**
All subjects (n:85)	*R*-value	0.872	0.691	0.857
	*p*-value	<0.001	<0.001	<0.001
Control (n:65)	*R*-value	0.762	0.603	0.774
	*p*-value	<0.001	<0.001	<0.001

#### 3.2.1. 8-picture recall

**Figure 3:**
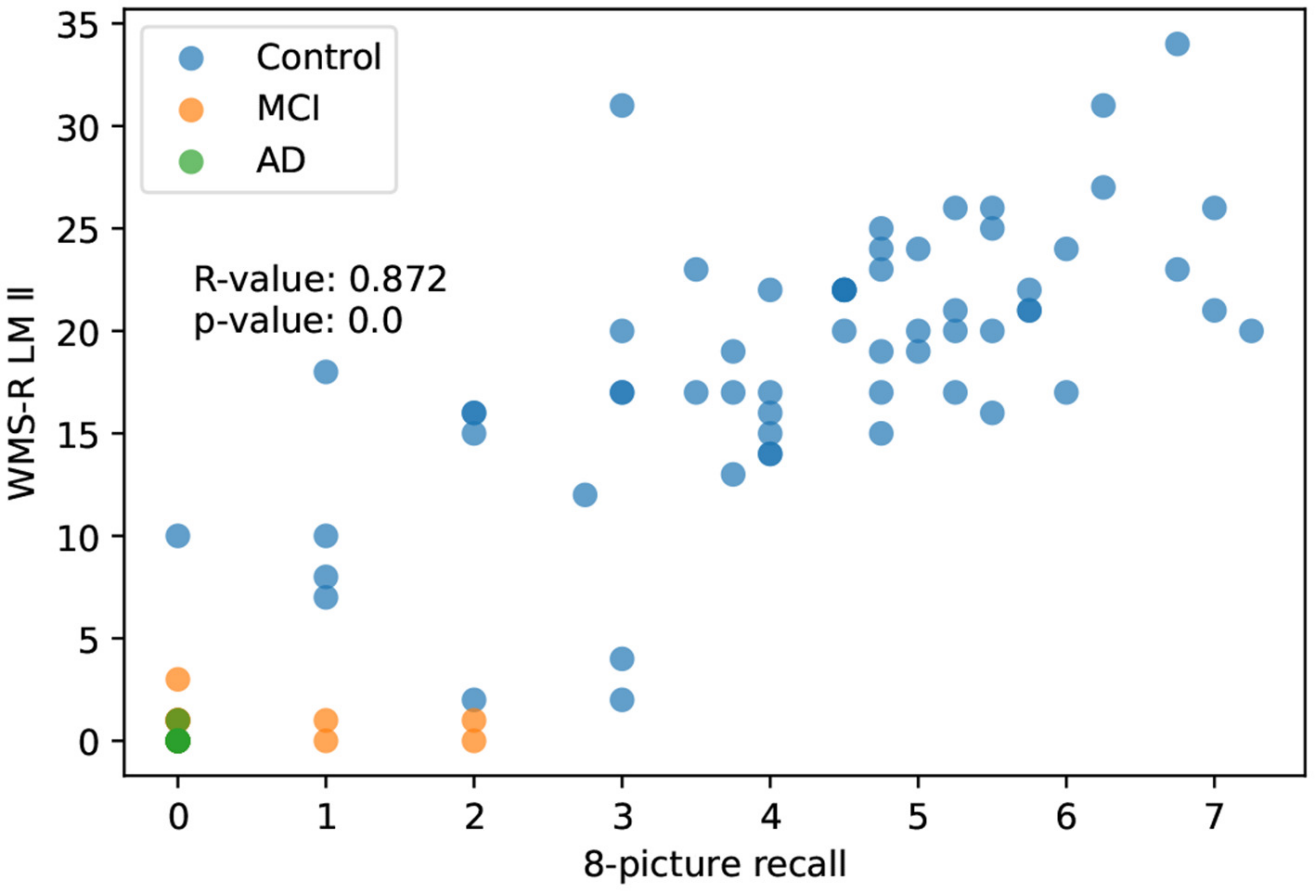
The correlation between 8-picture recall and WMS-R delayed recall.

The correlation between 8-picture recall and LM II score was *R* = 0.872, which is the highest correlation observed in our study and this correlation is preserved (*R* = 0.762) even when the analysis was limited to the control group (*p* < 0.001; Figure 3).

#### 3.2.2. 16-word recognition

**Figure 4:**
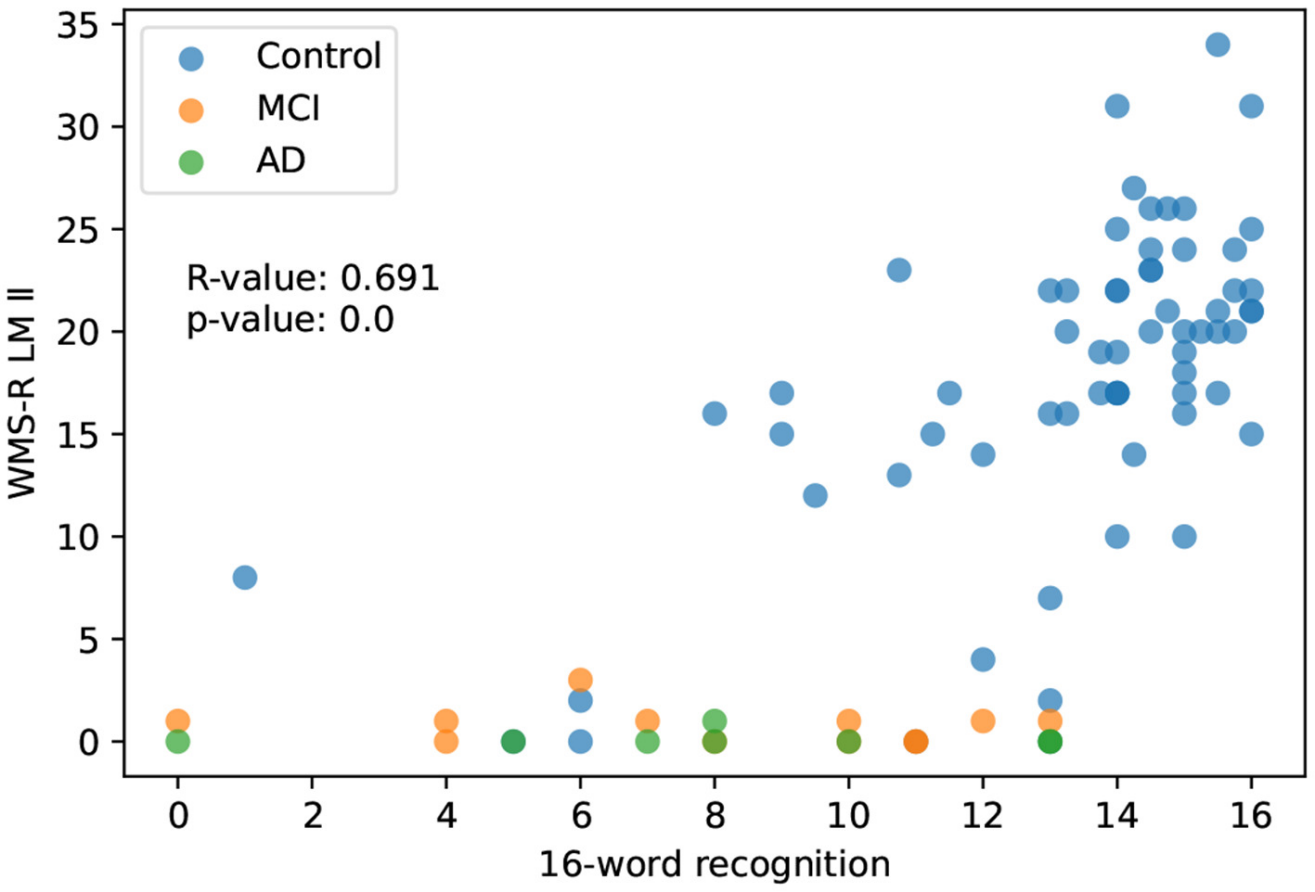
The correlation between 16-word recognition and WMS-R delayed recall score.

The correlation between 16-word recognition and LM II score was *R* = 0.691(*p* < 0.001). This association was also statistically significant in the normal control group alone (*R* = 0.603, *p* < 0.001; Figure 4). It is obvious that subjects with a 0 score of LM II have diverse scores broadly at 16-word recognition. This result is caused most likely by false recognition, in which a high score can be counted even when all responses are “yes.”

#### 3.2.3. Index of 8-picture recall and 16-word recognition as SAMS (Self Assessment Memory Scale)

**Figure 5:**
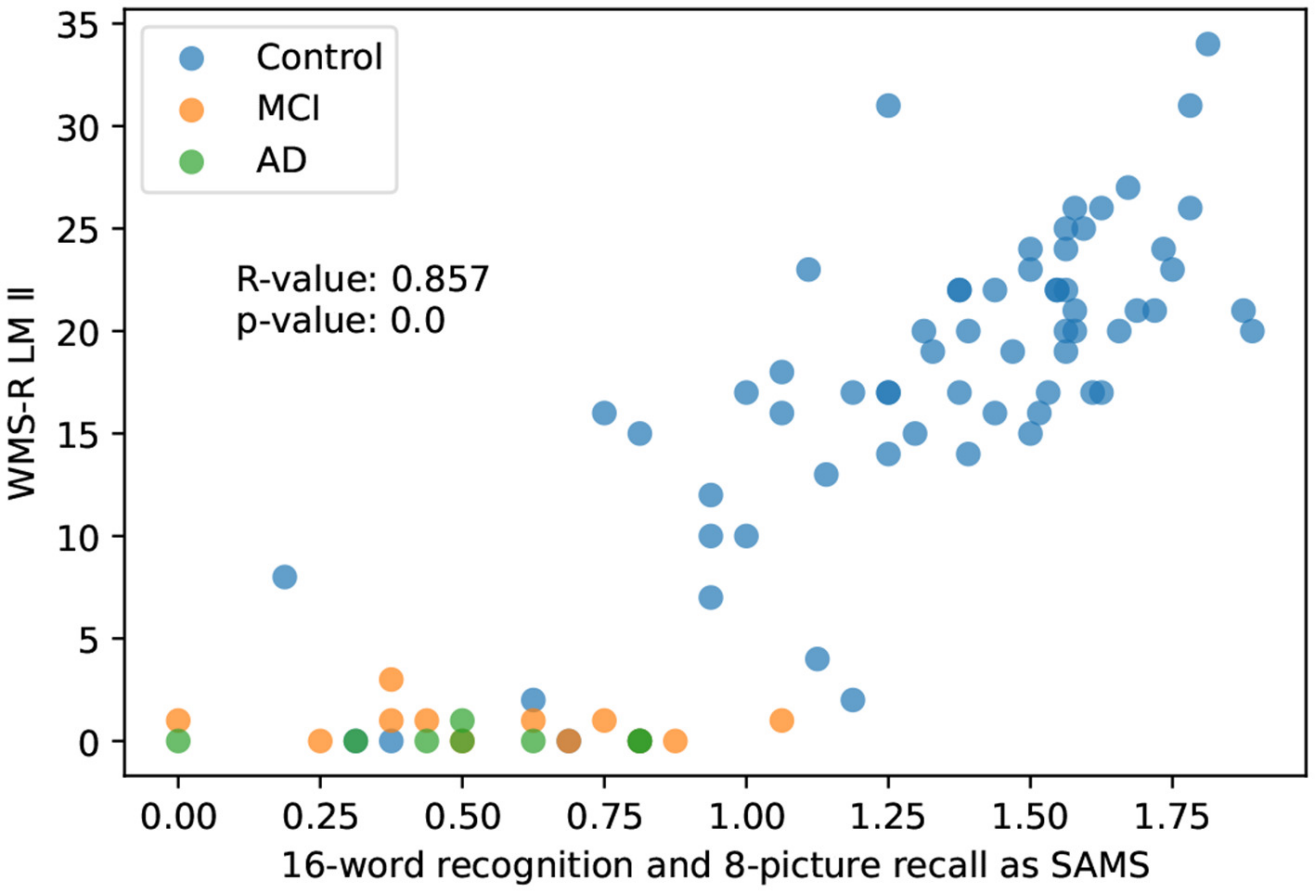
The correlation between the set of the two tasks and the WMS-R delayed recall.

In the primary task, the index score obtained as the sum of the percentage of correct answers in the two tests (8-picture recall and 16-word recognition) showed the highest correlation with the LM II score. In the main task, the correlation between the index from the two tasks and the LM II score was *R* = 0.857 (*p* < 0.001), showing the second highest correlation. In addition, when the analysis was limited to the control group, the highest correlation was observed (*R* = 0.774), suggesting that this index might be the best indicator for the early detection of memory loss (Figure 5).

## 4. Discussion

We have developed two methods as SAMS (Self Assessment Memory Scale) for detecting the early change in memory function, an 8-picture recall test and score of a 8-picture recall test score and a 16-word recognition test score. Both of these scores are well correlated with the LM II score of WMS-R, which indicates that our new method can easily predict the logical memory II score in about one-third of the time required by conventional methods.

In developing a new tool for estimating memory function, we aimed to develop a method that has a high correlation with the LM II score, which has been widely used (Petersen et al., 2010; Iwatsubo et al., 2018), and can be evaluated in a short time. Therefore, we selected a method with a higher *R*-value by correlation analysis. Our method is not intended for the diagnosis, but rather as a tool to easily assess whether or not there is a change in recall ability at home, and to encourage patients to seek medical attention if a change is observed. Since the LM II is an evaluation battery that is also used in general practice, an evaluation tool that ensures its correlation with the LM II is likely to facilitate the provision of memory functions to the physician in charge of the patient.

The method established in this study is capable of detecting changes in the recall, the first change observed in mild cognitive impairment (Aggarwal et al., 2005; Yaffe et al., 2006). Therefore, it was anticipated that a suitably difficult task would be required. Indeed, in a preliminary examination of a cohort of healthy subjects, the correlation between an easy task such as 5-word playback and LM II score was too low (*r* = 0.12). The LM II correlations improved for more difficult tasks [8-picture recall (*r* = 0.36), 12-word recognition (*r* = 0.31), and 16-word recognition (*r* = 0.46)]. Based on these results, we employed these two methods, 8-picture recall and 12-word recognition test.

In the study of 85 cases in which the subjects were extended to MCI and AD, the 8-picture recall showed a strong correlation with the LM II, *R* = 0.872 (*n* = 85). On the other hand, the scores for the 16-word recognition task resulted in a lower correlation with the LM II. This is because this task may have been higher than the true ability due to the false recognition of words that were not there. In Alzheimer's disease, it is said that memory impairment does not correlate with false recognition (Watson et al., 2001). However, we should originally consider the impact of the calculation of d-prime on the false recognition score (Tanner and Swets, 1954). In the present study, we did not have the necessary data for this calculation, so we could not conduct this study. This should be considered in the next validation study of this method.

Both the SAMS, which was calculated by summing the percentage of correct responses in each of the two tests (the highest value was 2), and the single test of 8-picture recall had a high correlation coefficient with LM II (*R* = 0.857 vs. 0.872), making it difficult to assign superiority or inferiority. However, in the control group (*N* = 65) excluding AD and MCI, the *R*-value of the 8-picture recall and the SAMS dropped with *R* = 0.762 and *R* = 0.774, respectively. This suggests that the SAMS score is more useful for subtle memory loss detection in the early phase.

Cogstate (Maruff et al., 2013), Mild Cognitive Impairment Screen (MCIS) (Shankle et al., 2005), and CogEvo (Ichii et al., 2020) have been commercialized as simple cognitive scales using digital devices. All of these methods have shown correlations with ADAS score and/or MMSE score because they evaluate multi domain cognitive functions, and do not focus on the evaluation of memory recall ability. Therefore, we believe that these methods are unsuitable to capture changes in cognitive function at the very early stage. On the other hand, the SAMS method reflects a high correlation with the LM II of WMS-R and is likely to capture changes in the early phases of Alzheimer's disease. The results suggest that when the SAMS score is less than 1, the WMS-R LM II score is also low and a medical examination in the hospital should be recommended. The relationship between SAMS score and amyloid accumulation (meaning preclinical AD) will be investigated in future studies.

The limitations of this study are that it was conducted at a single institution and that the number of subjects was large for the control group but small for the AD and MCI groups. This was because the control group was recruited from a community cohort and volunteers, and the AD and MCI groups were recruited from hospital outpatient clinics and nursing homes. However, this method is used to enable rapid and easy estimation of WMS-R logical memory II and is not a tool for diagnosing AD or MCI; therefore, the results obtained in this study were considered meaningful.

Since this method can be implemented on an electronic device such as smartphones and tablet computers by integrating with a speech recognition system, it is likely to contribute to the regular assessment of cognitive function at home and in the community. If the SAMS score showed below 1.0, he or she will be recommended to see a doctor at a memory clinic because his or her score of WMS-R LM II score is supposed to be below ten. We have just started developing such kinds of devices, and we are going to test their usefulness and validity as well as determination of cutoff values for each age group in the next study.

## Data availability statement

The original contributions presented in the study are included in the article/supplementary material, further inquiries can be directed to the corresponding author.

## Ethics statement

The studies involving human participants were reviewed and approved by the Ethics Committee of the Graduate School of Health Sciences, Kobe University. The patients/participants provided their written informed consent to participate in this study. Written informed consent was obtained from the individual(s) for the publication of any potentially identifiable images or data included in this article.

## Author contributions

HK and MU conceived the idea of the study and drafted the original manuscript. HK, MU, and MH developed the statistical analysis plan and conducted statistical analysis. HK and RO contributed to the interpretation of the results. AO supervised the conduct of this study. All authors reviewed the manuscript draft and revised it critically on intellectual content.

## Funding

This study received funding from Omron Healthcare Co., Ltd. The funder had the following involvement with the study: data collection. The funder was not involved in the study design, analysis, interpretation of data, the writing of this article, and the decision to submit it for publication.

## Conflict of interest

The authors declare that the research was conducted in the absence of any commercial or financial relationships that could be construed as a potential conflict of interest.

## Publisher's note

All claims expressed in this article are solely those of the authors and do not necessarily represent those of their affiliated organizations, or those of the publisher, the editors and the reviewers. Any product that may be evaluated in this article, or claim that may be made by its manufacturer, is not guaranteed or endorsed by the publisher.
